# Stage 2 Registered Report Personal factors and group creative outcomes: A correlational meta-analysis

**DOI:** 10.12688/f1000research.145939.1

**Published:** 2024-08-07

**Authors:** Adrien A. Fillon, Fabien Girandola, Nathalie Bonnardel, Jared B. Kenworthy, Lionel Souchet

**Affiliations:** 1 ERA chair of science and innovation Policy & Studies, University of Cyprus, Nicosia, 6015, Cyprus; 2 Department of Social Psychology, Aix-marseille University, Aix-en-Provence, France; 3 Department of Cognitive Psychology, Aix-Marseille University, Aix-en-Provence, France; 4 Department of Psychology, The University of Texas at Arlington, Arlington, Texas, USA

**Keywords:** brainstorm, creativity, meta-analysis, personality, correlation

## Abstract

Previous research has indicated that individual differences play a role in group creativity. Group creativity activities have different outcomes, leading to numerous ways to assess the effectiveness of these creative activities. To date, no meta-analysis has been performed on the relationship between the outcomes of the creative activity and personal factors of the group members. In this Registered Report, we conducted a meta-analysis ( *n* = 11, *k* = 72) on the relationship between personal factors and group creativity outcomes. We found weak support for a positive correlation between self-efficacy and group creativity outcomes, between *r* = .04 and *r* = .67. We found weak support for a moderation effect of time constraint, with stronger relationships for conditions limited to 20 minutes as opposed to 10 minutes. Finally, we found that only a few studies could be included in the meta-analysis, because many studies (1) did not directly measure creativity, or (2) measured other, less common personal factors. We call for a more systematic and direct approach to measuring creativity and an improvement of open science practices in the field. Data and analysis can be found at https://osf.io/xwph9.

The influential Yale study ( Taylor et al., 1958) was the first to test the performance of brainstorming techniques in idea generation. Since then, group creativity activities have been extensively studied. A group creativity activity can be defined as a group activity designed to develop numerous ideas that are original and useful ( Paulus & Nijstad, 2003; Taggar, 2019). In most cases, group creativity activities are based on explicit and elaborate instructions. For example, the brainstorming activity ( Meadow et al., 1959) has four explicit rules (i.e., go for quantity, withhold criticism, welcome wild ideas, combine and improve ideas). Brainwriting ( VanGundy, 1984) adds a fifth rule, as you must write down your ideas before sharing them with other group members. Other variants of brainstorming have also been proposed, for example, based on instructions to incite participants to evoke constraints related to the problem at hand, in addition to ideas ( Bonnardel & Didier, 2020). Creative Problem Solving (CPS, Osborn, 1963) also has four rules (i.e., clarify, generate ideas, solve, and implement). Other names than ‘brainstorming’ can be used for group creativity activities such as collaborative idea generation or idea generation groups ( Kenworthy et al., 2020). In this case, the instructions are generally brief, sometimes just a demand to “try to be as creative as possible,” but they give rise to generally poorer creative performance than more elaborated instructions ( Niu & Liu, 2009). Generally, a group creative activity involves the generation of original and useful ideas, with a standard instruction to not criticize ideas, to say whatever ideas came to mind, to focus on generating as many ideas as possible, to build on ideas, and to stay focused on the task ( Coursey et al., 2018; Osborn, 1963; Putman & Paulus, 2009). The most typical outcomes examined in group creativity research are the number, the originality (sometimes called novelty), and the usefulness (sometimes called relevance) of the ideas generated. While the number of ideas corresponds to a direct measure of quantity, the originality and usefulness consist of measures of quality, and in most cases, external raters assign numerical values to the ideas. These idea ratings are then averaged within the groups to produce a group quality score ( DeRosa et al., 2007).

Personal factors of group members are important to our understanding of how and why creative groups succeed in generating novel ideas and favoring innovation. At the individual level, several studies and meta-analyses have been conducted to study how personal factors, such as personality, affect creativity ( Lebuda et al., 2021; Litchfield et al., 2017). At the group level, some studies have indicated that the composition of the group can affect creative outcomes (e.g., Moreland et al., 1996). Since then, personality traits and cognitive trait differences have been studied in group creativity activities (for a review, see Coursey et al., 2018), but to our knowledge, no meta-analysis has been conducted regarding the link between personal factors and outcomes of group creative activities.

In this paper, we seek to answer two critical questions: (1) Which creative outcomes in group activities are associated with which personal factors; and (2) What are some potential moderators of the relationship between group creativity outcomes and personal factors?

Toward this end, we performed a correlational meta-analysis to contribute to the literature on the association between creativity outcomes at the group-level and personal factors of members within the group. A theoretical account of these two constructs is presented first, followed by our hypotheses on why and how the two constructs may be connected. Then, we will describe the procedure for conducting this meta-analysis and the results it allowed us to obtain.

## Personal factors

Extensive research has been conducted on personal factors influencing individual creativity, but less research has been done on people in social contexts ( Reiter-Palmon & Kaufman, 2018). Creativity was first investigated as an individual process, and teamwork was considered as a facilitator or inhibitor of individual creativity ( Amabile, 1996; Reiter-Palmon et al., 2012). Today, team creativity is central to product design and development, and to the solution of challenging problems. Understanding how each member of the creative group can have a different impact on the outcome of creative thinking is crucial since it can contribute to improving the process and customizing the creative activity for the participants.

For a long time, the empirical literature examining creativity activities mostly associated personal factors with personality traits ( Barron & Harrington, 1981; McCrae & Costa, 1995; Puryear et al., 2017; Yao & Li, 2021). Recently, researchers have started examining the effects of emotional disposition and cognitive differences to get a broader comprehension of individual differences and their contribution to group creative outcomes.

## Relationships between personal factors and group creativity outcomes

There is a growing interest in the link between personal factors and group creativity outcomes. Many existing studies have been conducted regarding the personal factors involved in fluency and flexibility ( Butler et al., 2003), originality and usefulness ( Baer et al., 2008), and number of ideas ( Brown et al., 1998). However, there have been debates regarding the relationship between personal constructs and group creativity outcomes due to mixed findings. Coursey et al. (2018) provided a review of individual factors in group creativity activities. In their overview, they stated (p. 26-27):

“It is presumed that there will be some degree of similarity between the effects observed for individuals and groups. However, we have highlighted a number of ways in which the effects of individual differences may not be simply additive. […] Thus, in agreement with the configural perspective of Moynihan and Peterson (2004), certain team compositions may be ideal for groups that go through the full phases of the creative process. One strategy would be to compose a team that had the ‘right mixture’ for the multiple phases, or one could compose separate teams specifically for different phases.”

Indeed, in a group setting, some social traits can improve creativity potential, for example, traits leading group members to be more attentive to the ideas of others, to process the shared ideas including the more radical ones, to be more motivated and persistent in the search for new ideas, to build on the ideas of others, and to share new ideas. We have summarized the list of personal factors we considered in this meta-analysis in Table 1.

**Table 1.** Commonly used measures of personal factors in creative groups.

Main term	Definition	Literature	Description of results
Personality trait	(Based on Costa & McCrae, 1992, p. 5-6)		
Openness to experience	“The term to refer to a broader constellation of traits. High-O individuals are imaginative and sensitive to art and beauty and have a rich and complex emotional life; they are intellectually curious, behaviorally flexible, and nondogmatic in their attitudes and values.”	Schilpzand et al. (2011, p. 67)	“As expected, we found that openness to experience was important for creative team outcomes.”
Extraversion	“a broad group of traits, including sociability, activity, and the tendency to experience positive emotions such as joy and pleasure.”	Jung et al. (2012, p. 30)	“In the first experiment, extraverts outperformed introverts in computer-mediated groups. In the second experiment, we exposed participants in computer-mediated groups to four levels of idea stimulation ranging from none to extremely high. Extraverts generated more unique and diverse ideas than did introverts in moderate- and high-stimuli conditions only.”
Conscientiousness	“Conscientiousness is a dimension that contrasts scrupulous, well-organized, and diligent people with lax, disorganized, and lackadaisical individuals.”	Baer et al. (2008, p. 274)	“In addition to demonstrating that teams composed primarily of individuals high on extraversion, high on openness, or low on conscientiousness were highly creative when team creative confidence was high, our results also indicated that composing teams mainly of high neuroticism or of low agreeableness members had little effect on team creativity.”
Agreeableness	“Agreeableness is primarily a dimension of interpersonal behavior. High-A individuals are trusting, sympathetic, and cooperative; low-A individuals are cynical, callous, and antagonistic”	Taggar (2002, p. 323)	“An individual's extraversion, conscientiousness, and agreeableness are positively associated with intragroup process behavior (team creativity-relevant processes at the individual level). In the regression equation, about 31 percent (p < .001) of the variation in intragroup process behavior was explained. Beta weights showed that conscientiousness contributed mostly to explaining team creativity- relevant processes at the individual level, followed by extraversion and agreeableness”.
Neuroticism	“The individual's tendency to experience psychological distress.”	Baer et al. (2008, p. 260)	“The above arguments suggest that teams composed of members who are likely to criticize others’ ideas and to provide candid feedback, that is, individuals high on neuroticism, should possess the potential to experience creative synergies.”
Emotion			
Social anxiety	“Been nervous or feeling discomfortable in a social context ( Leary & Kowalki, 1993)”.	Camacho & Paulus (1995, p. 1078)	“The results reported suggest that when social anxiousness is minimized, group brainstorming can be nearly as productive as nominal group brainstorming. Our results thus suggest that interactive brainstorming may be best suited for people who are low in social anxiety.”
Emotional intelligence	“The ability to monitor one’s own and others’ feelings and emotions, to discriminate among them, and to use this information to guide one’s thinking and actions”. ( Wang, 2015, p. 325)	Wang (2015, p. 340)	“The present study […] showed that average member EI increased elaboration, which in turn led to better performance in the informationally diverse condition.”
Cognition			
Cognitive style	“Individuals who tend to stick to a topic are known as convergent thinker. Individus who are more likely to free-associate and jump between topics are known as divergent thinkers.” ( Brown et al., 1998, p. 498)	Brown et al. (1998, p. 519)	“When a divergent thinker changes from a divergent partner to a convergent partner, their output (spoken ideas) increases. When a convergent thinker changes from a convergent partner to a divergent partner, their output decreases.”
Need for Closure	“a desire for a definite answer to a question, any firm answer, rather than uncertainty, confusion, or ambiguity” ( Chirumbolo, 2005, p. 61)	Chirumbolo et al. (2005, p. 74)	“The need for cognitive closure exerts an adverse effect on creativity in groups. Specifically, groups composed of individuals high (vs. low) in need for closure revealed a lower degree of productivity across multiple measures of group creativity. High (vs. low) need for-closure groups exhibited significantly lesser fluency of ideas.”
Creative Self-efficacy	“The capacity judgement about creative endeaviors” ( Richter et al., 2012, p. 1283)	Richter et al. (2012, p. 1287)	“The positive relation between [creative self-efficacy] and creativity was stronger in teams with greater shared [knowledge of who knows what].”
Epistemic Motivation	“Group members’ epistemic motivation—their willingness to expend effort to achieve a thorough and rich understanding of the world, including the group task or decision problem at hand” ( Bechtoldt et al., 2010, p. 623)	Bechtoldt et al. (2010, p. 633)	“We proposed that group creativity improves when members have high rather than low epistemic motivation. With regard to creative fluency—the number of nonredundant ideas and insights generated by the group— our hypothesis was supported in all three tests.”
Need for Cognition	“The tendency for an individual to engage in and enjoy effortful thinking” ( Huang and Liu, 2021, p. 2)	Huang and Liu (2021, p. 1)	“Psychological safety climate and the need for cognition were positively associated with team creativity through information elaboration.”

To point out important findings in the literature, Feist (1998) asserted that creative individuals need to balance between social stimulation and quiet reflection. Therefore, extroverted people may show better creative performance in some groups, depending on the need to interact with others (see also Anderson et al., 2008). Conscientiousness, which leads people to adhere to norms and rules ( Roberts et al., 2009), may help generate a high number of ideas, but not necessarily ideas that are original or useful. This idea is supported by Feist (1998), who found that artists, who need to develop original ideas individually, were generally low in conscientiousness. Agreeableness is a strong predictor of team performance because it is related to trust and morale ( Hogan et al., 1994). However, creative people are generally low in agreeableness ( Bechtoldt et al., 2012; Karwowski & Lebuda, 2016). Low neuroticism could be a more beneficial trait to have in a group creativity setting ( Bell, 2007; Da Costa et al., 2015; Peeters et al., 2008). Other personal factors can have differential links with various creativity outcomes. Emotional factors can play a role ( Kuška et al., 2020), such as anxiety ( Camacho & Paulus, 1995), and emotional intelligence ( Wang, 2015). It is also the case for cognitive dispositions such as cognitive orientation or cognitive styles ( Brown et al., 1998), and other cognitive traits such as creative self-efficacy ( Taggar, 2019; Tierney & Farmer, 2002), epistemic motivation ( Bechtoldt et al., 2010), Need for Closure ( Chirumbolo et al., 2004, 2005), and Need for Cognition ( Wu et al., 2014). For example, Need for Closure, a cognitive tendency to avoid ambiguity, is related to the quality of ideas generated but not originality ( Watts et al., 2017). Most studies mentioned above examined the relationships of interest at the individual level. At the time of writing, there is no literature review or meta-analysis on the relationship between personal factors and creativity outcomes in a group setting.

## Research aims and Hypotheses

In this study, we sought to examine: (1) the overall relationship between personal factors and creativity outcomes in group settings; and (2) moderators of these relationships.

## Moderators

We examined different moderators in the relationships between personal factors and group creativity outcomes, including environmental influence on how the activity was conducted, tasks factors related to the rules of the creative activity, and personal factors such as demographic variables. Our moderator hypotheses were exploratory and mostly based on the last review available on the subject ( Coursey et al., 2018). In this review, researchers indicated that there were very few studies on the subject, and we expected some moderators to be untestable meta-analytically in the absence of primary-level empirical data. We explored and reported all available relationships. In italics below are the hypotheses for which the literature on the subject tends to provide evidence for a relationship.

**Familiarity**

Familiarity with the group is the extent to which group members know each other ( Sosa & Marle, 2013). For example, familiarity can range from participants who do not know each other in a laboratory setting to teammates who have already worked with each other for a long time. In the study conducted by Sosa and Marle (2013), it was found that the more familiar group members are to one another, the better the creative outcomes. We hypothesized that individuals who struggle with novelty will produce better group creativity outcomes if they are familiar with the other group members. These groups include participants high in introversion ( Orengo Castellá et al., 2000), Need for Closure ( Chirumbolo et al., 2004), and social anxiety ( Camacho & Paulus, 1995).

*Familiarity*: In a non-familiar context, introversion, need for closure and social anxiety are negatively associated with creative outcomes. In a familiar context, the negative correlations are weaker than in a non-familiar context.

**Skill and knowledge diversity**

The idea behind skill diversity is similar to familiarity: people who are closer to each other tend to bond easier, leading to less perceived stress and a more positive social climate, resulting in better creative outcomes. At the same time, synergy can be difficult in an overly homogeneous group, because not every member adds creative value beyond the others ( Nijstad & De Dreu, 2002). A group with members who have substantial overlap in skills and knowledge may have limited creativity due to a lack of diversity. We exploratively tested the moderation hypothesis that skill and knowledge diversity in a group modifies the relationships between personality traits and creative outcomes.

**Group demography**

Diversity with respect to hierarchical status, gender, age, and field of study, leads groups to be more creative ( Choi, 2007; Paulus & van der Zee, 2015). In small groups, and using electronic brainstorming, research indicated that groups composed of women showed greater fluency (i.e., produced more ideas) than mixed groups, groups composed of men, or “solo” groups ( Peter et al., 2021). For group creativity, competition had a positive effect on creative outcomes for groups composed of men, but a negative effect for groups composed of women ( Baer, 2013). We explored the hypothesis that group demography modifies the relationships between personality traits and creative outcomes.

**Constraint**

Constraint refers to the degree of freedom in creative activity. Two major types of constraints can be implemented in brainstorming activities: production blocking, which prohibits members from sharing their ideas as they come to mind, and asynchrony, which means that participants generate ideas individually before sharing them with each other. In the production blocking condition, individual factors are less important for creativity ( Nijstad & Stroebe, 2006) than in the non-production blocking condition, whereas in the asynchronous condition, individual factors are more important than in the synchronous condition ( Paulus & Kenworthy, 2018).

*Constraint*: In a production blocking setting, the relationships between individual factors and creative outcomes are lower than in a non-production blocking setting.

In an asynchrony setting, the relationships between individual factors and creative outcomes are higher than in a synchrony setting.

**Type of task**

The type of task can influence the relationship between personal factors and creative outcomes. In conjunctive tasks, creative tasks in which participants pass ideas from one to another, the performance of the group is most strongly influenced by persons with traits that are highly positively or negatively related to creative outcomes. In disjunctive tasks, where participants share ideas before their selection, the influence of each individual is weaker because they do not influence the sharing process ( Coursey et al., 2018). We explored the evidence for an effect of this type of task in creative processes.

*Type of task*: In disjunctive tasks, the relationships between personal factors and creative outcomes are smaller in magnitude (regardless of sign) than in conjunctive tasks.

**Creative phase**

Harvey (2013) found that the diversity of ideas was positively related to divergent creativity (i.e., the mental process leading to producing ideas that are different, varied, and original), and negatively to convergent thinking (i.e., the mental process leading to narrowing the set of ideas generated towards a solution). Furthermore, convergent thinkers performed better in the convergent phase than in the divergent phase; divergent thinkers performed better in the divergent phase than in the convergent phase. As hypothesized by Coursey et al. (2018), introverts could be better at building and integrating creative ideas in the convergent phase, while extroverts could be less inhibited and make more contributions during the divergent phase.

*Creative phase*: Divergent thinking and extraversion are more strongly and positively associated with creative outcomes in the divergent phase than in the convergent phase.

Convergent thinking and introversion are less negatively associated with creative outcomes in the convergent phase than in the divergent phase.

**Number of participants**

The number of group members is critical in creating, sharing, and transforming ideas and information into projects. The number 5 is generally admitted as optimal in terms of maximizing interacting group performance ( Steiner, 1972). As the number increases, the creative performance of the group decreases ( Fellers, 1989). The problem is that, as the number of participants increases, the likelihood of dysfunctional behaviors also increases (i.e., dominance by individual members, fear of personal evaluation, fear of speaking in public, pressure for conformity, and task restrictions, see Fellers, 1989). For personal factors, the number of participants might “dilute” the creative contribution of each member, leading to a weaker (positive or negative) relationship. Dugosh et al. (2000) also found that high levels of off-task communication were detrimental in face-to-face brainstorming groups, and that off-task communication increases with the number of participants (particularly extrovert participants).

*Number of participants*: the higher the number of people who participate in the activity, the weaker the (negative or positive) relationships between personal factors and creative outcomes.

**Time pressure**

The possibility to create and share information depends on the time available. Most creative tasks are structured and time-limited, mostly because creativity is mentally effortful. Time limitation is detrimental to group creativity ( Karau & Kelly, 1992). Chirumbolo et al. (2004) found that time pressure reduced the percentage of creative acts during a group discussion and was positively related to personal need for closure (both reduced creative outcomes).

*Time pressure*: the negative relationship between need for closure and creative outcomes in group creativity is stronger under time pressure than under no pressure.

**Leadership**

It is challenging to assess how the leadership type will influence the relationship between personal factors and creativity. We decided to split leadership into two traditional types: transformational and transactional. In transformational leadership, the leader clearly states the goal and pushes the group toward attaining this goal. In transactional leadership, the leader relies on an exchange process in which group members are rewarded for accomplishing specific goals ( Jung, 2001; Mumford et al., 2019). Research has found that transformational leadership leads to higher creative outcomes than transactional leadership ( Jung, 2001; Sosik & Cameron, 2010; Zhang et al., 2011). Sosik and Cameron (2010) indicated that transformational leadership was related to an increase in motivation to create more ideas and ideas that are more original. On the contrary, Taggar (2019) explained that a cohesive team might follow the dominant actors in the team instead of trying to find more ideas. Thus, he hypothesized that excessively strong cohesion in a team following a (transformational) leader could impair creative collective efficacy. Anderson and Fiedler (1964) also showed that groups with participatory leaders (i.e., transactional leaders) had the highest number of ideas generated, and groups with supervisory leaders had the most original and useful ideas. We hypothesized that the leadership type has an effect on the relationship between personality and creative outcomes. Leaders close to the team who create a non-judgmental climate could help improve the performance of anxious, introverted, and less motivated members. On the other side, leaders with a more distant relationship with the group, in the exchange process to attain the goal and who do not contribute to the task, could reduce the performance of these members, reducing the global creative performance. We explored the hypothesis that the type of leadership influences the relationships between personality traits and creative outcomes, leading to giving more weight to the indication from Sosik and Cameron (2010) that transformational leadership leads to a stronger positive link or to the explanation of Taggar (2019) that transactional leadership leads to a stronger positive link.

**Publication status**

We examined publication status for possible moderating effects on the relationship between personal factors and creativity outcomes. Several recent meta-analyses ( Mathur & VanderWeele, 2020; Moreau & Gamble, 2020; Schmucker et al., 2017; Vosgerau et al., 2019) have suggested that including unpublished work can help improve the capture of the ‘true’ effect size. Accordingly, we expected that studies that were published are likely to report stronger associations than those that remained unpublished.

*Publication status*: published studies report stronger negative and positive relationships than unpublished studies.

## Methods

### Open Science Disclosures

We shared all procedures, materials, datasets, and analysis code on the Open Science Framework ( https://osf.io/xwph9/). The pre-registration and additional information about decisions are available in the supplementary materials of the extended data. We deviated from Stage 1 with the following: We did not use Scopus because we were not able to access it. We modified keywords during the literature search to have more results, as our first search was too restrictive. Finally, we have not conducted all publication bias tests mentioned, and the cumulative test because of the lack of studies to conduct them.

### Design

**P** **ersonal factors** are explained in Table 1. They consist of *personality traits*: 1) openness, 2) conscientiousness, 3) extraversion, 4) agreeableness, 5) neuroticism; *emotion*: 1) social anxiety and 2) emotional intelligence; *cognition*: 1) Cognitive style, 2) epistemic motivation, 3) self-efficacy, 4) need for closure, 5) Need for Cognition. The three categories of **creative outcomes** are 1) number of ideas generated, 2) originality of these ideas and 3) usefulness of the ideas.

### Eligibility criteria

Studies including personal factors (see Table 1) and measuring creative outcomes in group settings are included in our analysis.

### Search strategy

*Database searches.* To identify articles that are potentially relevant to our topic of investigation, we conducted searches using Google Scholar, Psychinfo, Web of Science - social science citation index, Proquest- dissertations and theses (for suitability, see Gehanno et al., 2013; Martin-Martin et al., 2019; Moreau & Gamble, 2020).

For personal factors, the keywords were personality trait*, openness, extraversion, introversion, conscientiousness, agreeability, neuroticism, anxiety, social anxiety, thinking style, convergent thinking, divergent thinking, Need for Closure, creative self-efficacy, epistemic motivation, Need for Cognition, emotional intelligence. For creative outcomes, the keyword was simply “creativity.” Initially, we had planned to use more specific search terms (such as “number of ideas”); however, this led to very few results which prompted us to switch to a broader search strategy. All search patterns included the following operators: “group*” OR “team” AND “correlation” *.*

During the search, keywords related to constructs were linked with the Boolean logic operators “OR” and keywords between construct 1 and construct 2 with “AND”. Variations of the keywords were included in the search with the original keywords if search results yielded fewer than 100 results, linked with “OR”. (e.g., “personality trait*” AND “useful*”). More information on the search pattern process can be found in the coding sheet under the tab “search pattern.” Database searches for each search pattern were terminated after combing through 30 records consecutively without potentially relevant papers for the inclusion criteria.

The search included papers listed under the “related articles” and “cited by” features in Google Scholar to identify papers that are similar or have cited the identified articles. We looked at other articles that were published by identified authors in the field to check if there were relevant papers that we may have missed. We systematically contacted the authors of the identified articles (see the pre-registered email template in the supplement) and issued a call for unpublished findings on ResearchGate and Twitter to find relevant unpublished data. For all the articles, titles, abstracts, tables, and methods sections were scanned to identify the relevance of a source.

### Inclusion and exclusion criteria

Correlational meta-analyses typically exclude studies that manipulated the target variable prior to measurement (e.g., Chevance et al., 2019), or alternatively conduct a separate meta-analysis for studies with manipulations or interventions (e.g., Schmitt et al., 2014; van Kleeck et al., 2010). First, we decided to restrict our meta-analysis to correlational studies that measure personal factors in creative contexts. Studies were excluded if they 1) experimentally manipulated an independent variable related to personal factors (e.g., manipulated motivation, social climate and anxiety, information and Need for Closure, etc.), 2) failed to report the crucial statistics necessary for a meta-analysis (i.e., correlation coefficient or other effect sizes that can be transformed into correlation coefficient, sample size), or 3) were not written in English or French unless all necessary information was provided in English or could be obtained from the authors.

### Screening

**Figure 1.**
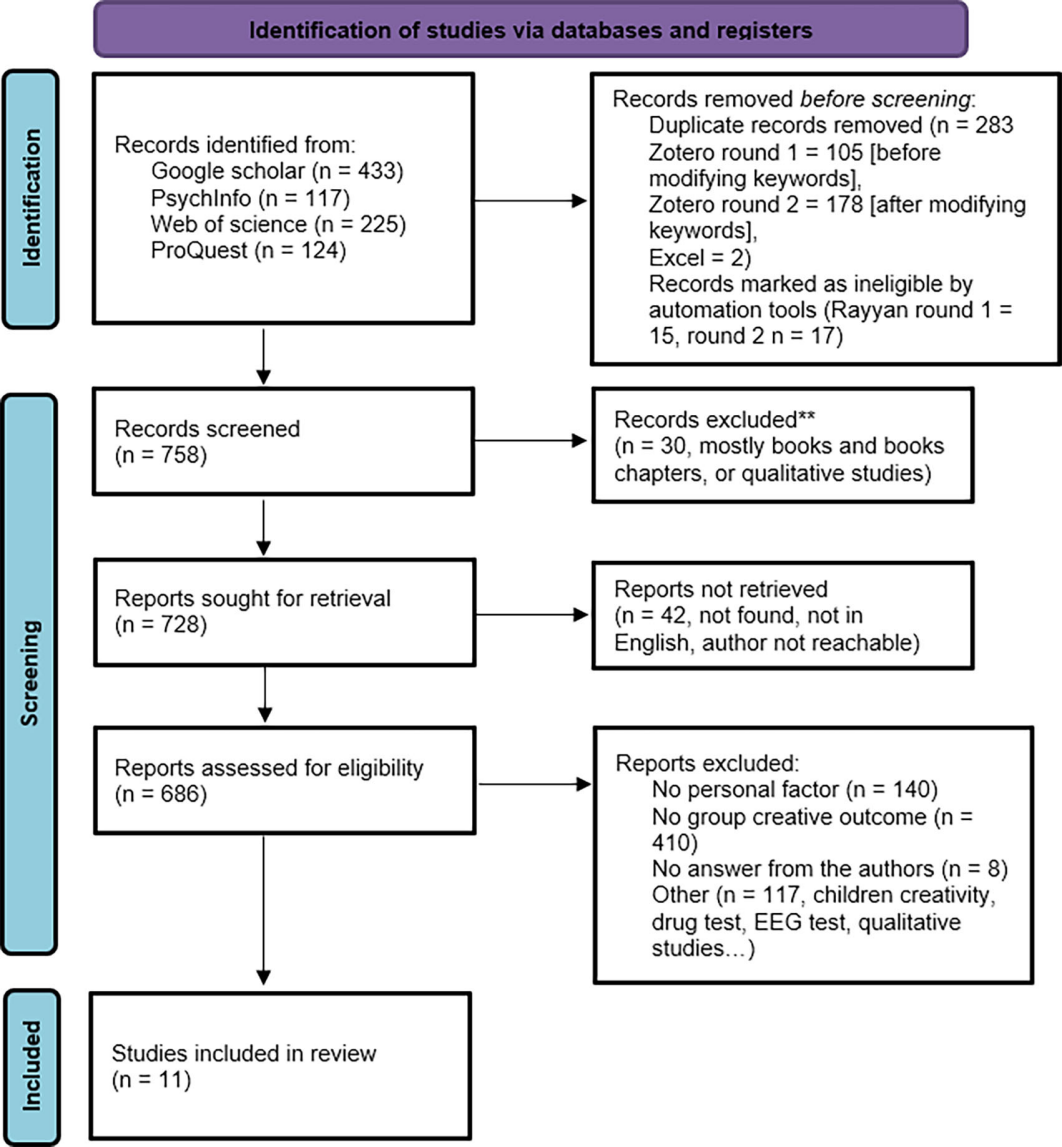
Meta-analysis flow diagram in accordance with PRISMA 2020 ( Page et al., 2020).

Studies that met our criteria were coded into the “Searched articles” tab within the coding sheet. Articles were scanned to determine whether they should be included into the main coding sheet or not. Reasons for exclusion were documented. Authors of studies with missing statistics were contacted for relevant datasets/information through the “contacting author” tab and the corresponding mail template (see supplementary material). If the dataset was provided, we included the article in the main coding tab. Finally, the PRISMA diagram in Figure 1 and the included studies in Table 2 summarize how and which studies were included.

**Table 2.** All studies/datasets included in the meta-analysis.

No.	Article	N	Personal factors measured	Creative outcome(s)	Publication status	Country
1	Morgan (1996)	90	Need for cognition	Number of Ideas	Published	USA
2	London (2006)	160	Self-efficacy, anxiety	Number of Ideas, originality	Published	USA
3	Brophy (1995)	375	Need for cognition	Number of Ideas	Published	USA
4	Mutlu (2017)	135	Big Five	Number of Ideas, Originality	Published	UK
5	He (2019)	109	Self-efficacy	Originality, Usefulness	Published	China
6	Lee and Park (2020)	267	Big five	Number of Ideas, Originality and Usefulness	Published	Singapore
7	Bolin and Neuman (2006)	312	Big five	Number of Ideas, Originality and Usefulness	Published	USA
8	Homan et al. (2015)	202	Self-efficacy	Number of Ideas, Originality and Usefulness	Published	Germany
9	Kenworthy (2020)	168	openness, extraversion, conscientiousness, Need for cognition	Number of Ideas and originality	Published	USA
10	Gheorghe (2020)	159	Emotional intelligence	Number of Ideas, Originality and Usefulness	Published	Romania
11	Bechtoldt (2010)	108	Motivation	Number of Ideas, Originality and Usefulness	Published	Netherlands

### Coding

Extracted data from the included studies were recorded in the “main coding sheet” tab. When available, the main correlations between personal factors and creative outcomes were recorded, along with intercorrelations between creative outcomes, the type of scale used, sample demographics, and publication information. Moderator variables were coded in a separate column in the main coding sheet.

### Analysis

We developed a Rmarkdown Script for the statistical analyses. The packages used are indicated in the supplementary material. Our main package for meta-analysis is Psychmeta ( Dahlke & Wiernik, 2019). We used Pearson’s correlation coefficient *r* as the main indicator of effect size. Whenever available, we used correlations obtained directly from authors of original papers. If only regression results were available, we converted to correlation by using a transformation provided in the supplement. If not possible, we asked the authors to provide either the correlation or raw data. Correlations were corrected for attenuation by using the formula ( Dahlke & Wiernik, 2020):

$$r_{c}=\frac{r_{\mathrm{obs}}}{\sqrt{r_{\mathrm{xx}\prime }}\sqrt{r_{\mathrm{yy}\prime }}}$$

Given the range of different outcomes for each category, we expected the heterogeneity in the sample to be relatively high. Thus, a random effect model was used for all relationships. Split conditions due to moderators were collapsed to allow for comparison of the relationships. All conversions and coding decisions were documented. A meaningful association is expected as having a correlation of at least *r* = .10 ( Cohen, 1988; Gignac & Szodorai, 2016; Schäfer & Schwarz, 2019). We documented all conversions and coding decisions. We included the original quotes and/or table/page numbers from the original articles into the coding sheet to facilitate reproducibility. We plotted forest plots presenting the correlation for every relationship. We presented the relation with confidence intervals and the sample size of each study.

Statistical heterogeneity between studies was determined using an 80% credibility interval (as used in Borenstein et al., 2009; Wiernik et al., 2017). Wiernik and Kostal (2019) explained how the credibility interval performed better than the most used *Q* significance test. The main reasons are that the *Q* test is underpowered in most situations and that it confounds the sample size of studies and the magnitude of effects found in the studies. Finally, Paterson et al. (2016) suggested thresholds for credibility intervals as an indication for moderation (e.g., corrected correlations < .15 as negligible, .15–.24 as small, .25–.39 as moderate, and ≥ .40 as large). If there was indeed meaningful heterogeneity, we explored potential moderators. Our design for the analysis is shown in Table 3.

**Table 3.** Study design.

Question	Hypothesis	Sampling plan	Analysis plan	Rationale for deciding the sensitivity of the test for confirming or disconfirming the hypothesis	Interpretation given different outcomes	Theory that could be shown wrong by the outcomes	Summary
What is the relationship between personal factors and creativity outcomes in group settings?	There will be a relationship between personal factors (big 5, emotional and cognitive traits) and creativity outcomes (originality, usefulness, number of ideas) in group settings.	Meta-analysis: we systematically collected all the relevant data available in various databases and asked for unpublished studies on Twitter and ResearchGate. We reported the sample size for every relationship.	We analyzed data with a psychometric meta-analysis using the psychmeta package ( Dahlke & Wiernik, 2019). The effect size is Pearson’s *r* corrected with internal consistency artifact distributions (alpha or composite reliability).	A meaningful association is having a correlation of at least r = .10 (lower side of the credibility interval).	An association > .10 was interpreted as a meaningful association, an association < .10 was interpreted as no association or not enough data to draw a conclusion based on the number of studies and participants involved. In both cases, the lack of evidence was only indicated and not interpreted.	Theories about creativity were mostly investigated as individual processes. A lack of correlation would only indicate that this personal factor is less important in group settings than in individual creativity for this particular creative outcome.	To investigate relationships between a personal factor and a creativity outcome in a group setting, we conducted a correlational meta-analysis. An association higher than *r* >. 10 may indicate support for the association, leading to the conclusion that a particular individual factor is associated with creativity in a group setting.
What are the moderators of the relationships between personal factors and creativity outcomes in group settings?	Moderators: Familiarity Skill Diversity Group demographics Constraints Task Type Creative Phase Number of Participants Time Leadership	Meta-analysis: we systematically collected all the data available in databases and asked for unpublished studies on Twitter and Researchgate. We reported the sample size for every relationship.	We added the moderator to the meta-analytic models.	We reported correlations for all relationships at a moderator level and compared the levels with a *Q* test based on the subgroups’ fixed effects.	A significant Cochran’s *Q* test determined if there was support for differences between levels of each moderator.	Most of our moderators’ hypotheses are exploratory hypotheses ( Coursey et al., 2018). Thus, an absence of evidence was only seen as a hypothesis not to investigate further.	To investigate moderators for the relationship between a personal factor and a creativity outcome in a group setting, we conducted a correlational meta-analysis with the moderator included. A significant Cochran’s *Q* test can indicate support for a difference between levels of a particular moderator explaining how the context can influence the relationships between personal factors and creative outcomes.
Does publication status influence the relationships between personal factors and creativity outcomes?	Publication status influences the status of the relationships. Specifically, published studies are likely to yield larger effect sizes than unpublished studies.	Meta-analysis: we systematically collected all the data available in databases and asked for unpublished studies on Twitter and Researchgate. We reported the sample size for every relationship.	We added publication status to the meta-analytic model.	We intended to report the fixed meta-analytical effect sizes for published and unpublished studies.	A significant Cochran’s *Q* test determined if there was support for a difference between published and unpublished studies.	The purpose of this moderator is to flag possible publication bias and no theory is involved.	We conducted a moderator meta-analysis with the publication status. A significant Cochran’s *Q* test can indicate support for a difference between published and unpublished studies.

***Exploratory analyses***

Initially, we had planned to include additional possible moderators that emerged during the literature search. Due to the low number of studies, such additional exploratory analyses were not possible.

***Publication bias***

To address possible biases, we corrected for sampling error and measurement error, as indicated in the guidelines for psychometric meta-analyses ( Schmidt & Hunter, 2015). Reliability was corrected using internal consistency artifact distributions (alpha or composite reliability) compiled from studies included in the present meta-analysis. A summary of weighted mean internal consistency can be found in the supplementary materials. We had originally planned to conduct a sensitivity analysis ( Mathur & VanderWeele, 2020) with the use of cumulative meta-analysis but could not do so due to the lack of studies.

Finally, we conducted a PET-PEESE analysis ( Stanley & Doucouliagos, 2014) and a p-curve analysis ( Simonsohn et al., 2014).

## Results

Meta-analytic results are shown in Table 4. There were 25 combinations, and for 12 of them (48%), only one effect size was available, making it impossible to perform a meta-analysis. Of the studies available for meta-analysis, only three constructs suggest a positive non-zero relationship: the relationship between creative self-efficacy and the number of ideas generated (ρ = .35, 95%IC [.04, .67]) the originality of ideas (ρ = .35, [.09, .60]), and the usefulness of ideas (ρ = .49, [.33, .65]).

**Table 4.** Summary of Meta-Analysis findings.

Personality trait	Creativity outcome	*k*	*N*	$\bar{r}$	*SD*r	*SD*res	$\bar{\rho }$	SDrc	SD	95% CI	80% CR
Openness	Number of ideas	4	879	.18	.15	.14	.22	.19	.17	[−.08, .52]	[−.05, .50]
	Originality	4	879	.00	.19	.18	.00	.24	.22	[−.38, .39]	[−.36, .37]
	Usefulness	1	267	.14	—	—	.18	—	—	[ .03, .34]	[—, —]
Conscientiousness	Number of ideas	4	879	−.06	.15	.13	−.07	.17	.15	[−.35, .21]	[−.32, .18]
	Originality	4	879	−.10	.07	.03	−.12	.09	.03	[−.26, .02]	[−.17, −.06]
	Usefulness	1	267	−.09	—	—	−.11	—	—	[−.27, .04]	[—, —]
Extraversion	Number of ideas	4	879	.08	.13	.11	.10	.15	.13	[−.14, .33]	[−.11, .31]
	Originality	4	879	−.06	.08	.04	−.08	.09	.05	[−.23, .07]	[−.16, .00]
	Usefulness	1	267	−.12	—	—	−.15	—	—	[−.30, −.00]	[—, —]
Agreeability	Number of ideas	3	711	.03	.08	.05	.04	.10	.06	[−.22, .29]	[−.08, .15]
	Originality	3	711	−.02	.10	.07	−.03	.12	.09	[−.34, .28]	[−.20, .14]
	Usefulness	1	267	.06	—	—	.08	—	—	[−.08, .24]	[—, —]
Neuroticism	Number of ideas	3	711	−.03	.05	.00	−.04	.06	.00	[−.18, .10]	[−.04, −.04]
	Originality	3	711	−.06	.19	.18	−.08	.25	.23	[−.69, .53]	[−.52, .35]
	Usefulness	1	267	−.07	—	—	−.09	—	—	[−.25, .07]	[—, —]
Social anxiety	Number of ideas	1	160	−.17	—	—	−.19	—	—	[−.36, −.02]	[—, —]
Emotional intelligence	Number of ideas	1	159	.01	—	—	.01	—	—	[−.18, .21]	[—, —]
	Originality	1	159	.09	—	—	.12	—	—	[−.08, .32]	[—, —]
	Usefulness	1	159	.05	—	—	.07	—	—	[−.14, .28]	[—, —]
Self-efficacy	Number of ideas	2	352	.30	.03	.00	.35	.04	.00	[ .04, .67]	[ .35, .35]
	Originality	3	461	.28	.08	.03	.35	.10	.04	[ .09, .60]	[ .27, .42]
	Usefulness	2	301	.39	.01	.00	.49	.02	.00	[ .33, .65]	[ .49, .49]
Epistemic motivation	Number of ideas	1	108	.48	—	—	.51	—	—	[ .36, .66]	[—, —]
	Originality	1	108	.02	—	—	.02	—	—	[−.19, .23]	[—, —]
	Usefulness	1	108	.29	—	—	.33	—	—	[ .14, .53]	[—, —]

*Note: k* = number of studies contributing to meta-analysis; *N* = total sample size; $\bar{r}$ = mean observed correlation; SDr = observed standard deviation of r; SDres = residual standard deviation of r; $\bar{\rho }$ = mean true-score correlation; SDrc = observed standard deviation of corrected correlations (rc); SD = residual standard deviation of $\bar{\rho }$ ; CI = confidence interval around $\bar{\rho }$ ; CR = credibility interval around $\bar{\rho }$ . Correlations corrected using artifact distributions.

### Moderator analyses

Due to the extremely small number of effect sizes, it was not possible to analyze all moderators. Table 5 summarizes the moderator data.

**Table 5.** Moderators of the link between personal factors and group creativity.

Moderator	Information
Familiarity	*β* = -0.06, *p* = 0.25, no sign of an effect ( *k* = 52).
Skill and Knowledge Diversity	Only one study used this moderator (i.e. Brophy, 1995).
Group Demography	Only one study with diverse participants (i.e. Homan et al., 2015).
Constraint	No study with constraint
Type of Task	No study with conjunctive tasks
Creative phase	Only one study reported an effect size for the convergent phase (i.e., Brophy, 1995).
Number of participants	*β* = -0.01, *p* = .64, no sign of effect ( *k* = 72).
Time limit	*β* = 0.01, *p* = .01 weak but significant effect ( *k* = 60).
Leadership	One study on transformational leadership, no study on transactional leadership
Publication status	All included studies are published.
Gender	*β* = 0.00, *p* = .95, no sign of an effect ( *k* = 62).

In particular, only one moderator is significant, the effect of time ( *β* = 0.01, *p* = .01). However, the *β* estimator is small and indicates that each minute spent in the creativity session increases the relationship between personal factors and creative outcomes by *r* = .01. Additionally, the residual heterogeneity test is significant, indicating that other uncaptured moderators are at work ( *Q* = 382.19, *p* < .001).

### Inter-correlations

The inter-correlations between the creative outcomes provide no evidence for a relationship between the number of ideas and originality (7 studies, ρ = -.01, [-.30, .29]), a positive relationship between the number of ideas and usefulness (2 studies, ρ = .67, [.670, .674]), and originality and usefulness (2 studies, ρ = .47 [.10, .85]). For the personality traits, 4 studies found a weak relationship between openness and extraversion ρ = .18, [.02, .33] (see Table 6).

**Table 6.** Inter-correlations between creative outcomes, and between personal constructs.

First construct	Second construct	*k*	*N*	$\bar{r}$	$SD_{r}$	$SD_{\mathrm{res}}$	$\bar{\rho }$	$SD_{r_{c}}$	$SD_{\rho }$	95% CI	80% CR
Number of ideas	Originality	7	1 390	−.01	.32	.31	−.01	.32	.31	[−.30, .29]	[−.46, .44]
Number of ideas	Usefulness	2	351	.67	.00	.00	.67	.00	.00	[.67, .67]	[.67, .67]
Originality	Usefulness	2	351	.47	.04	.00	.47	.04	.00	[.10, .85]	[.47, .47]
Self-Efficacy	Social Anxiety	1	160	−.49	—	—	−.49	—	—	[−.61, −.37]	[—, —]
Openness	Conscientiousness	4	879	.15	.17	.16	.15	.17	.16	[-.12, .42]	[−.11, .40]
Openness	Extraversion	4	879	.18	.10	.07	.18	.10	.07	[.02, .33]	[.06, .29]
Openness	Agreeability	3	711	.11	.14	.13	.11	.14	.13	[−.25, .47]	[−.13, .35]
Openness	Neuroticism	3	711	.01	.16	.14	.01	.16	.14	[−.38, .41]	[−.26, .28]
Conscientiousness	Extraversion	4	879	.19	.24	.23	.19	.24	.23	[−.19, .58]	[−.19, .58]
Conscientiousness	Agreeability	3	711	.23	.19	.18	.23	.19	.18	[−.24, .71]	[−.11, .57]
Conscientiousness	Neuroticism	3	711	−.05	.36	.35	−.05	.36	.35	[−.93, .84]	[−.70, .61]
Conscientiousness	Need for Cognition	1	168	.42	—	—	.42	—	—	[.30, .55]	[—, —]
Extraversion	Agreeability	3	711	.19	.14	.12	.19	.14	.12	[−.15, .54]	[−.04, .43]
Extraversion	Neuroticism	3	711	−.11	.45	.44	−.11	.45	.44	[−.22, 1.00]	[−.94, .72]
Extraversion	Need for Cognition	1	168	.07	—	—	.07	—	—	[−.08, .22]	[—, —]
Agreeability	Neuroticism	3	711	−.02	.34	.33	−.02	.34	.33	[−.86, .82]	[−.64, .61]

*Note: k* = number of studies contributing to meta-analysis; *N* = total sample size; $\bar{r}$ = mean observed correlation; SDr = observed standard deviation of r; SDres = residual standard deviation of r; $\bar{\rho }$ = mean true-score correlation; SDrc = observed standard deviation of corrected correlations (rc); SD = residual standard deviation of $\bar{\rho }$ ; CI = confidence interval around $\bar{\rho }$ ; CR = credibility interval around $\bar{\rho }$ . Correlations corrected using artifact distributions.

### Power analysis

**Figure 2.**
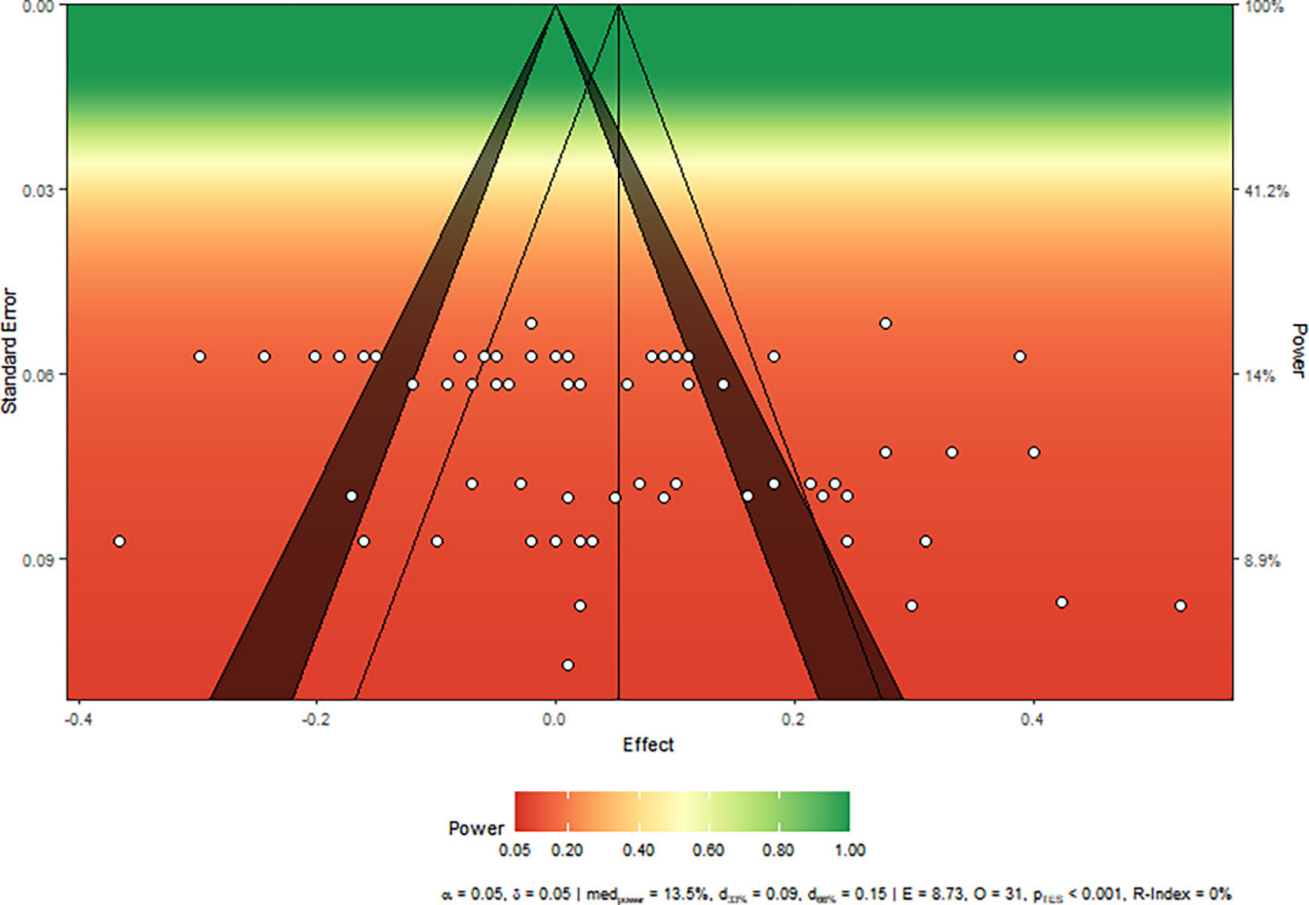
Power test of all studies.

We created a sunset plot in Figure 2 to display the statistical power of studies included in the meta-analysis. The average power is 13.5% and the replicability index 0% which means that we have no chance to reject H0 when there is a true effect, and no chance at all to replicate one study given the median average power of studies included in the meta-analysis and success rates of these studies (see Motyl et al., 2017 for R-index).

### Reliability

Table 7 presents the mean reliability scale of all variables included. The reliability was high across all variables, between.75 and.91.

**Table 7.** Mean reliability scale.

Variable	Artifact	*k*	*N*	*m*	*sd*	*sd.res*
Need for cognition	qxi_irr	2	465	.866	.035	.031
	rxxi_irr	2	465	.751	.061	.055
Self-efficacy	qxi_irr	3	461	.908	.027	.023
	rxxi_irr	3	461	.825	.049	.042
Agreeableness	qxi_irr	2	402	.865	.047	.043
	rxxi_irr	2	402	.750	.080	.074
Extraversion	qxi_irr	3	570	.915	.020	.016
	rxxi_irr	3	570	.838	.036	.029
Conscientiousness	qxi_irr	3	570	.905	.026	.022
	rxxi_irr	3	570	.820	.046	.040
Neuroticism	qxi_irr	2	402	.864	.008	.000
	rxxi_irr	2	402	.747	.013	.000
Openness	qxi_irr	3	570	.877	.033	.029
	rxxi_irr	3	570	.769	.059	.051
Number of ideas	qxi_irr	6	1 401	.945	.047	.046
	rxxi_irr	6	1 401	.896	.089	.088
Originality	qxi_irr	7	1 313	.907	.074	.072
	rxxi_irr	7	1 313	.827	.133	.131
Usefulness	qxi_irr	4	676	.871	.047	.044
	rxxi_irr	4	676	.761	.086	.080

*Note:* qxi_irr is the Restricted reliability index (indirect RR) and rxxi_irr is the Restricted reliability coefficient (indirect RR).

### Publication bias

The PET-PEESE analysis was not significant (PET: *t* = 1.95, *p* = .06; PEESE: *t* = 1.85, *p* = .07). In particular, the average correlation found by the PET test is *r* = -.17 [-.40, .06] and by the PEESE test *r* = -.06 [-.18, .06] which indicates an effect size close to 0 with a tendency to be rather negative.

**Figure 3.**
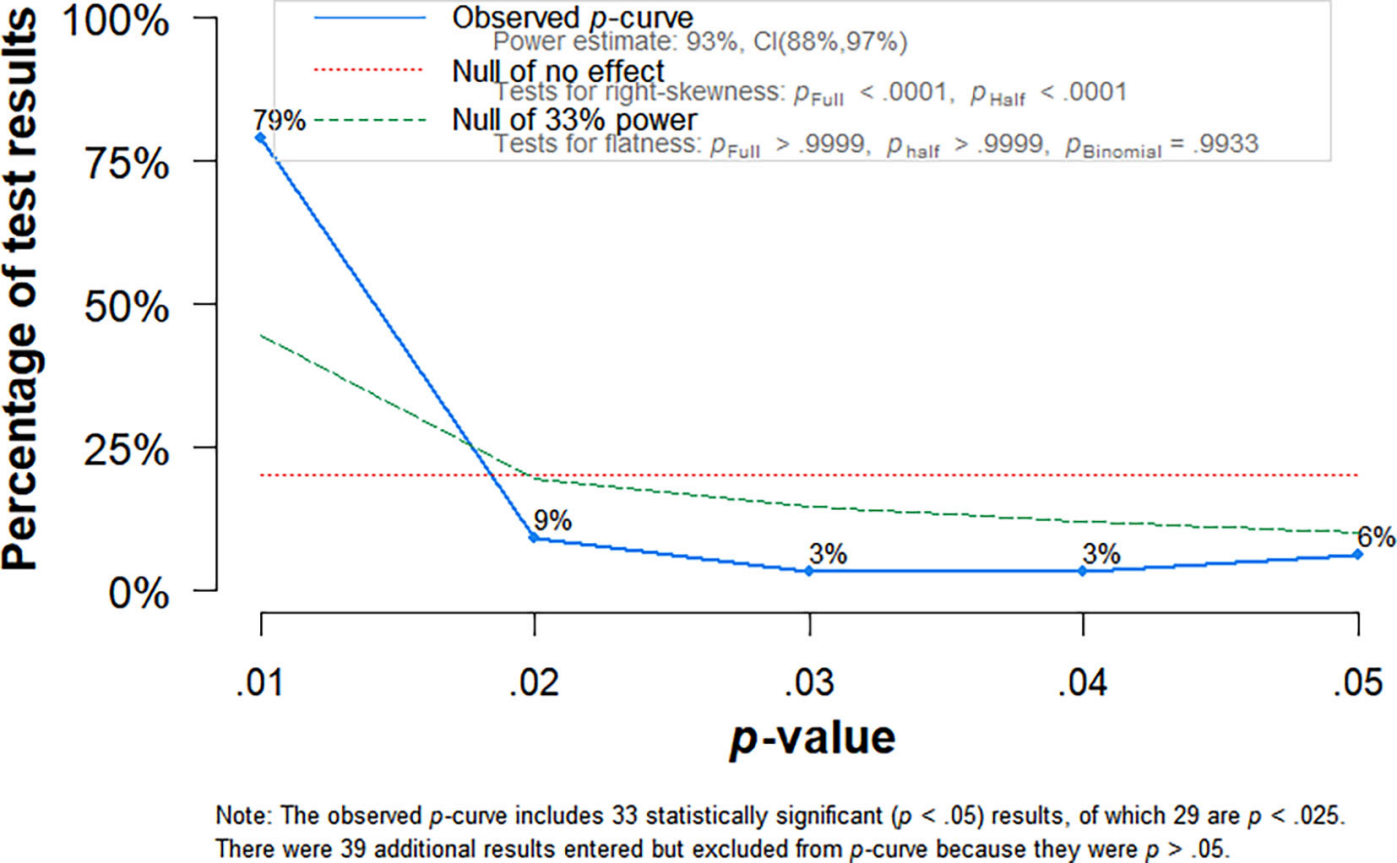
P-curve analysis.

The p-curve test indicated no signs of unpublished non-significant results (see Figure 3). It also indicated a statistical power of 93% [0.88, 0.97]. However, in the case where there are few effect sizes and strong heterogeneity, the p-curve test is not reliable ( van Aert et al., 2016).

Regarding the cumulative analysis, it was not possible to perform it because of the too small number of studies included in the analysis (< 20).

## Discussion

### Personal factors related to creativity in group setting

This meta-analysis investigated the links between personal factors and group creativity. The results are limited, given the very few studies and thus the very few effect sizes we could include. We found support for only one relationship, positive, between self-efficacy and all three creativity outcomes: the number, originality, and utility of ideas.

These results, although disappointing, are not so surprising. In 2018, Coursey and colleagues wrote (p. 2-3): “ *Whereas many studies have examined the role of individual differences in individual creativity, relatively few have examined the role of individual differences in group and team creativity.*” A little further on, they concluded ( Coursey et al., 2018, p. 26): “ *We have provided an overview of how individual difference factors may influence group creativity. Much of this is based on the literature concerning individual differences in individual creativity. It is presumed that there will be some degree of similarity between the effects observed for individuals and group*s.”

Our systematic review confirms that the majority of studies are based on individual creativity, as we found only 11 studies to include in our meta-analysis. Focusing on the creativity side, out of the 758 articles selected—with the criterion requiring the exact term “group” or the exact term “team” to be mentioned—160 studies concerned *individual* creativity, creative personality, perception of being creative, creative potential, or creative intelligence. Furthermore, when selecting the most used indicators in the creativity literature (number, originality, and usefulness of ideas), we found that the majority of studies did not measure group creativity with these indicators.

Seventy-two studies measured perceived creativity, whether by other team members or by the manager. In most of these studies, creativity was measured by Zhou and George’s (2001) 13-item scale, an example of which is “the team/team member suggests new ways to achieve goals or objectives” or “the team/team member often has new or innovative ideas”. The scale has not been psychometrically evaluated and there seems to be no evidence of a link between the scores on this scale and the actual creativity of individuals. We have not found any studies that measured both perceived creativity with this scale *and* group creative outcomes. In addition to this measure, about 20 studies measured ‘general team performance’, asking a question about creativity in this performance, or about creative decision-making in a team, but then measured only the choice made by the team. In contrast, direct measures of creativity were much rarer. This could be because such measures require experts in the creative area who can code ideas according to the indicators used (numbers, originality, and usefulness of ideas). Finally, despite the selection criteria, 181 results were not about creativity or did not measure it, with the word “creativity” being mentioned in the introduction or discussion of the study.

Furthermore, there was a small number of studies that manipulated individual factors, for example by measuring personality traits prior to experimentation, and by creating groups by separating individuals according to their level (low or high) on a personality trait. Others measured, for example, optimism, individualism, temperament, depression, envy, critical thinking, or self-constructs. In total, these exclusion criteria applied to 136 studies. It would be possible to conduct an experimental meta-analysis on the subject, which would not measure the relationship between individual factors and group creativity but the impact of a particular personality trait on group creativity. However, the results could be as disparate and inconclusive as in this meta-analysis, as other exclusion criteria would apply. Indeed, many of these studies also included only measures of perceived creativity, measures of individual creativity, and some were qualitative in nature. Thus, it is not clear that a meta-analysis of this type would provide enough effect sizes to conduct an informative analysis.

Out of the 11 studies included, 1 is from 2019, and 3 are from 2020, a sign that personality factors and group creativity are currently being investigated. In this sense, it is possible that this meta-analysis came a bit too early compared to the primary studies on the subject.

***Time limit***

The moderator analysis revealed a possible weak effect of time on the relationship between personal factors and creativity outcomes, as each minute spent in the creativity session increases the relationship between personal factors and creative outcomes by *r* = .01. However, given the limitation of the data, we could not test the hypothesis on cognitive closure. Most of the studies included in the analysis had a time constraint between 5 and 10 minutes, and for these studies, the moderation analysis revealed no effect. The effect of time limit was found for the studies comparing 10 and 20 minutes; the relationships were systematically stronger for the 20 minutes than for the 10 minutes condition. This finding suggests that a longer session leads to a stronger possibility for participants to express their individuality in group creative tasks, at least up to 20 minutes. We are limited in the generalizability of our findings, as the relationships were only found for 5 associations relating self-efficacy to originality/usefulness.

### Future research directions

The results of this meta-analysis may help to improve the field of research linking creativity and personality.

First, we built on the conjecture of Coursey et al. (2018) and improved current knowledge by systematically quantifying the data on the subject. We found that the available data was limited. Very few studies measure group creativity. Most studies measure perceived creativity, through questionnaires and surveys, and some studies make up their own measures of creativity, according to their own criteria. It would be possible to improve our knowledge of group creativity by using standardized creativity methods and protocols and by using direct measures, especially as in the majority of the studies included, inter-expert reliability was high, indicating that the perception of creativity is shared among experts.

Only one personal factor had a positive relationship with the three group creative outcomes: self-efficacy. This is not surprising, as self-efficacy is one of the most widely used individual factors in psychology. For example, the book *Self-efficacy: The exercise of control* ( Bandura, 1997) is cited 108 314 times on Google scholar (December 2022). The other social, cognitive, and emotional factors seem to be less studied or not studied in correlational studies. Surprisingly, many studies reported some personality traits, but not all. In the same way that some researchers use their own way of measuring creativity, others only partially measure personality (sometimes measuring types and not traits, or a single trait). This treatment of personal factors limits the possibilities of aggregating data in meta-analysis. The field could be improved by using standardized personality questionnaires and reporting all data points necessary.

Several studies measured the relationship between individual factors and group creativity, but as they did not report information essential for a meta-analysis, we could not include them. Of the 11 studies included, we requested the information for 6 of them. 8 additional studies could not be included because we were unable to contact the authors. Thus, we could have nearly doubled the number of studies included if information had been made available. The field of the research could be improved by systematically sharing the raw data and the analysis conducted. In addition to the benefit of the aggregation into a meta-analysis, this would allow for the conduct of individual participant data (IPD) meta-analyses ( van Aert, 2022).

A traditional meta-analysis is conducted at the group level - in other words, by asking the question “How do average group personal factors correlate with average group creativity?” An IPD meta-analysis can answer a more precise question: “How does an individual’s personality trait in this group correlate with his or her creativity in the group?” This question cannot be answered without data at the individual level.

The power of the studies was very low, around 13.5%. The number of participants is quite high (see Table 2), but the correlations seem to be extremely low, which requires an extremely high number of participants to indicate an effect if it exists. Again, reporting the raw data at the individual level would increase the precision of the estimates, improving the power of the study without the need to dramatically increase the number of participants.

The studies included were conducted in the USA, UK, China, Germany, Singapore, the Netherlands, and Romania. More importantly, many of the studies were performed on students doing group projects. It would be preferable to conduct studies in other contexts, including non-WEIRD, non-student samples. The many moderators that we tried to highlight were not investigated enough or were not investigated at all. We recommend that researchers methodically record all the data relating to the context of the creative activity, in particular the time allocated to participants, the type of task, the number of participants per group, and the individual characteristics of the participants (demographics, type of recruitment, randomization of the participants in groups or not, etc.).

### Interpretations and implications

One should be cautious in drawing a relationship between perceived creativity and actual creativity. For example, Pretz and McCollum (2014) did not find evidence for a relationship between self-rated and expert-rated creativity ( *r* = -.07). Professionals wishing to improve group creativity should use objective measures of creativity, or expert observations, rather than relying on personal or peer measures. This could be done through a common definition of originality and usefulness, followed by a pre-test rating by peers. To this objective, it is possible to define an intra-class correlation threshold for which the correlation is high enough to indicate that observers are homogeneously rating the originality and usefulness of ideas (typical thresholds are between 0.75 and 0.90, Koo & Li, 2016). Intraclass correlation is a type of correlation performed on groups of data, unlike Pearson correlation which operates on pairs of data (it is also called interclass correlation); however, the formulas used for both types of correlation are similar.

If the intra-class correlation is sufficient, then observers can continue to evaluate ideas. If the correlation is not sufficient, then it is necessary to find the cause of this lack of relationship, which may be related to a misunderstanding of the objective, difficulty in understanding the originality or usefulness of the ideas in relation to the creativity theme, or a difference in the appreciation of the ideas. After the exchange, the experts will carry out the analysis on a new sample until the intra-class correlation is sufficient to score all the ideas.

It seems important to increase the attention of researchers and professionals to individual differences in group creativity. Although it is difficult to make recommendations based on the meta-analysis results, the hypotheses stated remain possible. Fostering openness to new ideas, paying attention to factors promoting group dynamics (extraversion, agreeableness, low neuroticism, high emotional intelligence, low social anxiety, and low need for closure), and making sense of the common goal (motivation, need for cognition) remain possible avenues for improving creative outcomes in groups.

In particular, a conclusion can be made about creative self-efficacy. Self-efficacy was the only factor to show a positive relationship with group creative outcomes in the meta-analysis. One limitation of this relationship is that creative self-efficacy is not easy to develop. Several recent studies have failed to improve it ( Capron Puozzo & Audrin, 2021; Ohly et al., 2017). However, these studies had extremely small sample sizes ( *n* = 69 and *n* = 23 respectively).

It is also possible that there are several types of creative self-efficacy. Hughes et al. (2018) suggested that there are creative leaders and individuals who are more focused on a creativity support function. This leader/supporter distinction could be related to personality traits, but their relationship remains to be investigated.

The final finding concerns the environment associated with creativity. In this meta-analysis, only one moderating effect appears to be different from chance, namely time. This is a moderator that has been the subject of many studies on individual and team creativity. In particular, almost a third of the best ideas in terms of originality are generated in the first 5 minutes, and after 15 minutes, less than 10% of the ideas are of very good quality, while 50% of them are judged as bad ( Reinig & Briggs, 2008). It is possible that personal factors that promote persistence on task (e.g., motivation, conscientiousness, sense of self-efficacy) also promote the possibility of having original ideas longer, a hypothesis that remains to be tested. Furthermore, in terms of the relationship between constructs, one study ( Jung et al., 2015) indicated that people who generate the most ideas also come up with better quality ideas, with quality defined as an average between originality and usefulness. Another application of this type of study for professionals is to build on the most creative individuals to improve the overall creativity of all participants, regardless of their personal factors.

## Conclusion

This meta-analysis was an attempt to understand the relationship between individual factors and group creativity. It shows a link between self-efficacy and indicators of group creativity with a low level of evidence, and a small and positive moderation effect of time. During the search phase, we found that most studies concern individual creativity, and studies concerning group creativity do not directly measure creativity, but the perception of creativity by an internal or observing member of the group. Direct measures of creativity (number of ideas generated, originality, and usefulness) appear to be anecdotal, and the results found seem to mostly indicate a lack of relationship. Self-efficacy, a well-known individual factor in improving performance and learning ( Bandura, 1997), is positively associated with all of the group creative outcome measures. In other words, it appears that groups feeling they have the capacity to be creative are indeed creative, whether in terms of the number, originality, or usefulness of the ideas generated by these groups.

## Authorship declaration

Please see the table below.

**Contributor roles taxonomy.**

**Table:** 

Role	AF	FG	NB	LS	JK
Conceptualization	•	•	•	•	
Pre-registration	•				
Data curation	•				
Formal analysis	•				
Funding acquisition					
Investigation	•				
Pre-registration peer review/verification		•	•	•	•
Data analysis peer review/verification					•
Methodology	•				•
Project administration	•				
Resources	•				
Software	•				
Supervision		•	•	•	
Validation	•				•
Visualization	•				
Writing-original draft	•				
Writing-review and editing	•	•	•	•	•

*Note:* See https://www.casrai.org/credit.html for the details and definitions of each role.

## Data Availability

The underlying data can be found in Bonnardel et al. (2023). The data are shared under the license CC-BY 4.0. The project contains the following underlying data:
• The meta-analytical dataset (data and code > creativitymeta.xlsx) • Literature search folder (bibliographic data) The project contains the following underlying data:
• Supplementary.pdf (pre-registration information and additional information about decisions, and deviations from Stage 1) • The analysis script (data and code > ma-brainstorm-analysis. Rmd and moderator analysis. Rmd) • The result outputs and figures from the analysis script in the result folder. • The track-changed version of the second stage manuscript in the PCI-RR stage 2: tracked changes version. • The stage 1 files in the Stage 1 folder (data and code with randomized results, Main manuscript.pdf, output of the code, and a supplementary.pdf ) • The Main manuscript.pdf as accepted in Stage 2 by PCI.RR

## Software availability

Source code available from: https://github.com/adrien-fillon/Personal-factors-and-group-creative-outcomes/

Archived source code at time of publication: https://doi.org/10.17605/OSF.IO/XWPH9 in data and code > ma- brainstorm-analysis. Rmd and moderator analysis. Rmd

License: Apache License 2.0
