# Supplementary material for: Stage 2 Registered Report Personal factors and group creative outcomes: A correlational meta-analysis
Source: F1000Res. 2024 Aug 7;13:904. [Version 1] doi: 10.12688/f1000research.145939.1 (PMC12413614; doi:10.12688/f1000research.145939.1)
Supplement: Peer review history

# Reviewer report: Reviewer response for version 1

**DOI:** 10.5256/f1000research.159962.r401455

**Published:** 2025-09-05

**Authors:** Hamit Coskun

**Affiliations:** 1 Bolu Abant Izzet Baysal University, Bolu, Turkey

This manuscript addresses an important gap in the literature by presenting one of the first meta-analyses on the relationship between personal factors and group creativity. The registered report format and the commitment to open science practices (e.g., sharing data and analysis on OSF) are notable strengths that enhance the transparency and reproducibility of the study.

 That said, the meta-analysis is constrained by a relatively small number of included studies (n = 11, k = 72), which limits the robustness and generalizability of the findings. The reported effect sizes are mostly weak, and substantial heterogeneity in the measurement of creativity and personal factors across studies complicates the interpretation of results. A further methodological limitation is that many studies did not directly measure creativity, reducing the strength of the evidence base available for synthesis.

 Overall, the study makes a valuable contribution by highlighting methodological shortcomings in existing research and by calling for more systematic measurement approaches and broader datasets in future investigations. While the present findings should be interpreted with caution, the manuscript offers meaningful guidance for advancing research on group creativity and individual differences.

Is the work clearly and accurately presented and does it cite the current literature?

Yes

If applicable, is the statistical analysis and its interpretation appropriate?

Yes

Did the authors adhere precisely to the registered experimental procedures? If not, has an explanation been provided regarding any change?

Yes

Are all the source data underlying the results available to ensure full reproducibility?

Yes

Is the study design appropriate and is the work technically sound?

Yes

Are the data able to test the authors’ proposed hypotheses by satisfying the approved outcome-neutral conditions (such as quality checks, positive controls)?

Yes

Are the conclusions drawn adequately supported by the results?

Yes

Are the introduction, rationale and stated hypotheses the same as the approved Stage 1 submission?

Yes

Are any unregistered post hoc analyses added by the authors justified, methodologically sound and informative?

Yes

Are sufficient details of methods and analysis provided to allow replication by others?

Yes

Reviewer Expertise:

group brainstorming, creativity

I confirm that I have read this submission and believe that I have an appropriate level of expertise to confirm that it is of an acceptable scientific standard.

# Reviewer report: Reviewer response for version 1

**DOI:** 10.5256/f1000research.159962.r312033

**Published:** 2024-08-07

**Authors:** Julia Rohrer, Christopher Chambers

**Affiliations:** 1 Persönlichkeitspsychologie und Psychologische Diagnostik, Universitat Leipzig, Leipzig, Saxony, Germany; 2 School of Psychology, Cardiff University, Cardiff, Wales, UK

What determines whether groups of people can come up with ideas that are both original and useful? Since the 1960s, this question has been intensively studied with the help of more or less structured group creativity activities such as brainstorming or creative problem solving, with subsequent rating of the generated ideas.

 In this line of research, personal factors—such as personality traits, and other interindividual differences in emotion and cognition—have received substantial attention as potential correlates of creative outcomes of group activities. This has spawned a sprawling literature that, to date, has not yet been synthesized. Thus, empirical findings in this literature, which are also sometimes contradictory, have not yet been well-integrated.

 In the present study, Fillon et al. conducted the first meta-analysis of correlations between personal factors and group creativity outcomes. The authors searched and synthesized the existing published literature according to predetermined criteria to (1) assess the overall relationship between a broad list of personal factors and creativity outcomes in group settings and (2) explore potential moderators of these relationships. 

 In total, 11 studies could be included in the meta-analysis. They provided weak support for a positive correlation between self-efficacy and the three investigated group creative outcomes, number of ideas, originality of ideas, and usefulness of ideas. With respect to moderators, many of the planned analyses could not be conducted due to the low number of studies. The only finding that arose was weak evidence for the idea that time constraint moderates associations: relationships between personal factors and group creativity outcomes were slightly stronger for tasks limited to 20 minutes rather than 10 minutes. Statistical power overall was low across studies.

 The authors conclude their meta-analysis with the observation that the available data on the topic are very limited. They suggest that to improve our knowledge of the topic, future studies should adhere to standardized creativity methods and protocols and implement expert ratings of creativity. They also call for increasing the availability of raw data in this field of study to improve the accumulation of knowledge about links between personal factors and the creative performance of groups.

 The Stage 2 manuscript was evaluated over one round of in-depth review provided by the recommender and Chris Chambers, as the original reviewers were no longer available. Based on additional changes to the manuscript, the recommender judged that the manuscript met the Stage 2 criteria and awarded a positive recommendation.

**URL to the preregistered Stage 1 protocol:** https://osf.io/nybg6

**Level of bias control achieved:** Level 3. *At least some data/evidence that was used to answer the research question had been previously accessed by the authors (e.g. downloaded or otherwise received), but the authors certified that had not yet observed ANY part of the data/evidence prior to IPA.*

Is the work clearly and accurately presented and does it cite the current literature?

Yes

If applicable, is the statistical analysis and its interpretation appropriate?

Yes

Did the authors adhere precisely to the registered experimental procedures? If not, has an explanation been provided regarding any change?

Yes

Are all the source data underlying the results available to ensure full reproducibility?

Yes

Is the study design appropriate and is the work technically sound?

Yes

Are the data able to test the authors’ proposed hypotheses by satisfying the approved outcome-neutral conditions (such as quality checks, positive controls)?

Yes

Are the conclusions drawn adequately supported by the results?

Yes

Are the introduction, rationale and stated hypotheses the same as the approved Stage 1 submission?

Yes

Are any unregistered post hoc analyses added by the authors justified, methodologically sound and informative?

Yes

Are sufficient details of methods and analysis provided to allow replication by others?

Yes

Reviewer Expertise:

NA

We confirm that we have read this submission and believe that we have an appropriate level of expertise to confirm that it is of an acceptable scientific standard.
